# Estimated glucose disposal rate and risk of stroke and mortality in type 2 diabetes: a nationwide cohort study

**DOI:** 10.1186/s12933-021-01394-4

**Published:** 2021-10-06

**Authors:** Alexander Zabala, Vladimer Darsalia, Marcus Lind, Ann-Marie Svensson, Stefan Franzén, Björn Eliasson, Cesare Patrone, Magnus Jonsson, Thomas Nyström

**Affiliations:** 1 Department of Clinical Science and Education, Karolinska Institutet, Södersjukhuset, 11883 Stockholm, Sweden; 2 Institute of Medicine, University of Gothenburg, Gothenburg, Sweden; 3 Department of Medicine, NU Hospital Group, Uddevalla, Sweden; 4 Centre of Registers in Region Västra Götaland, Gothenburg, Sweden; 5 Department of Molecular Medicine and Surgery, Karolinska Institutet, Stockholm, Sweden; 6 Department of Vascular Surgery, Karolinska University Hospital, Stockholm, Sweden

**Keywords:** Type 2 diabetes, Estimated glucose disposal rate, Insulin resistance, Stroke, All-cause mortality

## Abstract

**Background and aims:**

Insulin resistance contributes to the development of type 2 diabetes (T2D) and is also a cardiovascular risk factor. The aim of this study was to investigate the potential association between insulin resistance measured by estimated glucose disposal rate (eGDR) and risk of stroke and mortality thereof in people with T2D.

**Materials and methods:**

Nationwide population based observational cohort study that included all T2D patients from the Swedish national diabetes registry between 2004 and 2016 with full data on eGDR and categorised as following: < 4, 4–6, 6–8, and ≥ 8 mg/kg/min. We calculated crude incidence rates and 95% confidence intervals (CIs) and used multiple Cox regression to estimate hazard ratios (HRs) to assess the association between the risk of stroke and death, according to the eGDR categories in which the lowest category < 4 (i.e., highest grade of insulin resistance), served as a reference. The relative importance attributed of each factor in the eGDR formula was measured by the R^2^ (± SE) values calculating the explainable log-likelihoods in the Cox regression.

**Results:**

A total of 104 697 T2D individuals, 44.5% women, mean age of 63 years, were included. During a median follow up-time of 5.6 years, 4201 strokes occurred (4.0%). After multivariate adjustment the HRs (95% CI) for stroke in patients with eGDR categories between 4–6, 6–8 and > 8 were: 0.77 (0.69–0.87), 0.68 (0.58–0.80) and 0.60 (0.48–0.76), compared to the reference < 4. Corresponding numbers for the risk of death were: 0.82 (0.70–0.94), 0.75 (0.64–0.88) and 0.68 (0.53–0.89). The attributed relative risk R^2^ (± SE) for each variable in the eGDR formula and stroke was for: hypertension (0.045 ± 0.0024), HbA1c (0.013 ± 0.0014), and waist (0.006 ± 0.0009), respectively.

**Conclusion:**

A low eGDR (a measure of insulin resistance) is associated with an increased risk of stroke and death in individuals with T2D. The relative attributed risk was most important for hypertension.

**Supplementary Information:**

The online version contains supplementary material available at 10.1186/s12933-021-01394-4.

## Introduction

The prevalence of type 2 diabetes (T2D) is increasing worldwide with rising human suffering from morbidity and mortality [1]. People with T2D are at higher risk of cardiovascular disease compared to people without diabetes, and a high prevalence of atherosclerotic disease i.e., myocardial infarction, peripheral artery disease and stroke exists [2, 3].

Strong traditional risk factors such as hypertension and hyperlipidaemia explain some of the increased risk of stroke observed in people with T2D. Recently our group showed that people with T2D with poor glycaemic control were at higher risk for stroke compared with T2D people who were well controlled demonstrating the importance of glycaemic control and the association for stroke [4]. Insulin resistance, which is more or less obligate in T2D and often following hyperglycaemia, is also suggested to be a strong risk factor for cardiovascular disease [5]. Preventing insulin resistance would yield as much as 40% reduction in atherosclerotic disease regardless of other well known risk factors such as hypertension, hyperlipidaemia, hyperglycaemia or obesity, involved in the insulin resistance state [6].

Gold standard technique measuring insulin resistance is the euglycaemic hyperinsulinaemic clamp method [7]. However, it is invasive and costly and therefore not suitable for large-scale clinical use. For that reason, a method based on the readily available clinical factors waist circumference, hypertension, and glycosylated haemoglobin A1c (HbA1c) was developed to estimate glucose disposal rate (eGDR) in patients with type 1 diabetes [8]. This method is proven to have a high precision when compared to the euglycaemic hyperinsulinaemic clamp method, and therefore, an excellent tool to measure insulin resistance in a large patient population [9]. Recently, our and other groups have used eGDR as a proxy for insulin resistance to predict long-term outcomes also in people with T2D [10].

Whether grade of insulin resistance predicts stroke in people with T2D is not well known [11, 12]. We performed a nationwide population-based cohort study using the Swedish national diabetes register (NDR) to investigate the association between insulin resistance measured by eGDR and risk of first-time stroke and mortality thereof in people with T2D.

## Methods

This was a nationwide registry based retrospective nationwide cohort study, approved by the regional ethics committee in Gothenburg, Sweden (EPN Dnr 776-14).

### Study population and data sources

All adult individuals (≥ 18 years) with T2D between 2004 and 2016 were included, from the Swedish national diabetes register (NDR) [13]. T2D was defined as diabetes treated with diet or oral hypoglycaemic agents alone, or age of > 40 years at onset of diabetes and treatment with insulin alone, or in combination with oral hypoglycaemic agents [14].

Data from NDR was combined with; the in-patient registry (IPR) with nationwide data for primary and secondary discharge diagnoses [15], the causes of death register, and the longitudinal integration database for health insurance and job market studies (LISA; Statistics Sweden) for socioeconomic data [16]. The linkage was made by utilising the Swedish national identity number ensuring a unique number sequence that all Swedish citizens are identified by.

### Estimated glucose disposal rate (eGDR)

The eGDR (mg/kg/min) was calculated as previously described according to the following formula: eGDR = 21.158 − (0.09 * WC)  −  (3.407 * HT)  −  (0.551 * HbA1c) [WC = waist circumference (cm), HT = hypertension (yes = 1/no = 0), and HbA1c = HbA1c (% DCCT)] [8].

Since the eGDR formula was developed for use in individuals with type 1 diabetes, we investigated the correlation between eGDR formula with a euglycaemic hyperinsulinaemic clamp procedure from 24 male T2D patients that have been investigated before in our lab (Additional file 1: Table S1). The correlation between clamp and eGDR were *r* = 0.73 (Additional file 1: Table S1; Figure S1 top).

Analysis of HbA1c was performed at local laboratories with the high-performance liquid chromatography Mono-S method and was quality-assured nationwide by regular calibration. We converted all HbA1c values to standard values according to the National Glycohaemoglobin Standardization Program [17]. Hypertension was defined as treatment with anti-hypertensive medication, or systolic blood-pressure > 140 mmHg, or diastolic blood-pressure > 90 mmHg, respectively.

### Study outcomes

A flow chart of the studied patients is presented in Additional file 1: Figure S2. The main outcome was time to stroke and mortality (divided in all-cause mortality and cardiovascular mortality). Patients were studied from index date until stroke, death or end of the study (Dec 31, 2016). International Classification of Diseases codes (ICD codes) I63 and I64 were used for ischaemic stroke, and I61 to I62 for intracerebral haemorrhage, to identify all strokes. Subarachnoid haemorrhage (ICD code I60) was not included in the study. Only the first hospital admission was used for patients with several stroke episodes. For the CV death outcome, the following ICD codes were used: I20-I25 and I61-I64. The ICD codes for comorbidities are given in Additional file 1: Table S2.

### Statistical analysis

The study cohort is described using standard descriptive statistics in terms of averages with standard deviation for continuous variables and counts with percentages for categorical variables. The incidence rate of all stroke (haemorrhagic stroke and ischaemic stroke), haemorrhagic stroke and ischaemic stroke was calculated as the number of events per 1000 person years and presented with 95% exact Poisson confidence intervals (CI).

The association between eGDR (mg/kg/min) was categorised as previously used cut-off levels [9, 10]: < 4 (highest grade of insulin resistance i.e., reference), 4–5.99, 6–7.99, and ≥ 8 mg/kg/min) and stroke events were illustrated using Kaplan–Meier curves and analytically assessed using three Cox regression models; unadjusted, adjusted for age and sex, and adjusted for age, sex, diabetes duration, LDL-cholesterol, HDL-cholesterol, triglycerides, lipid lowering medication, micro albuminuria, macro albuminuria, creatinine, estimated glomerular filtration rate (eGFR), retinopathy, smoking, physical activity, disposable income, marital status, education, cardiovascular morbidities, renal disorder, hyperglycaemia, amputation, dementia, psychiatric disorder and gastric bypass surgery.

The shape of the association between eGDR and stroke events was assessed using Cox regression models where the effect of eGDR was modelled using smoothing splines with 5 degrees of freedom in models also containing sex as a binary variable and a smoothing spline with 5 degrees of freedom modeling the effect of age.

The relative predictive performance for each variable in the eGDR formula was evaluated using Heller R^2^ [18].

The association between eGDR and post stroke mortality was assessed using three Cox regression models; unadjusted, adjusted for age at the index stroke event and sex, and adjusted for sex, age at the stroke event and the last pre-stroke observations on systolic blood pressure, diastolic blood pressure, body mass index (BMI) LDL-cholesterol, HDL-cholesterol, smoking, eGFR, lipid lowering medication and anti-hypertensive treatment. P-values below 5% are regarded as indicators of statistical significance and there was no adjustment for multiple comparisons. The statistical analysis was done using R 4.0.2 and SAS 9.4

## Results

### Study population and patient characteristics

A total of 104 697 individuals with T2D (women 44%) with a mean age of 62.9 years, and with a diabetes duration of 4.1 years had a baseline eGDR from index date and were included in the study. After categorising individuals according to eGDR groups a total of 24,706 (24%), 40,187 (38%), 21,042 (20%) and 18,762 (18%) had an eGDR of < 4, 4–6, 6–8 and ≥ 8 mg/kg/min, respectively. The mean age, the proportion of men, diabetes duration, HbA1c levels, blood pressure, physical inactivity, proportion of individuals with cardiovascular disease, nephropathy all decreased with increasing eGDR. Also, individuals in lower eGDR categories were more often treated with antihypertensive treatments, lipid-lowering agents, oral glucose-lowering agents and insulin (Table 1).

**Table 1:** Baseline characteristics of 104 697 patients with type 2 diabetes mellitus categorised in four groups of estimated glucose disposal rate (eGDR)

	All n = 104 697	eGDR < 4 (mg/kg/min) n = 24 706	eGDR 4–6 (mg/kg/min) n = 40 187	eGDR 6–8 (mg/kg/min) n = 21 042	eGDR > 8 (mg/kg/min) n = 18 762
Age, years (SD)	62.9 (11.5)	62.3 (10.2)	65.3 (10.7)	62.0 (12.7)	59.5 (12.2)
Age at onset, yrs (SD)	58.8 (11.5)	57.6 (10.3)	60.9 (10.8)	58.3 (12.5)	56.1 (12.3)
Diabetes duration, years (SD)	4.1 (5.7)	4.7 (6.1)	4.3 (5.8)	3.6 (5.4)	3.5 (5.3)
Women, n/N (%)	46099/104697 (44.0)	8621/24706 (34.9)	17797/40187 (44.3)	10616/21042 (50.5)	9065/18762 (48.3)
Smoking, n/N (%)	16125/98754 (16.3)	3738/23150 (16.2)	5542/37932 (14.6)	3546/19903 (17.8)	3299/17769 (18.6)
Marital status, n/N (%)					
Married	57106/104697 (54.5)	12,762/24706 (51.7)	22573/40187 (56.2)	11082/21042 (52.7)	10689/18762 (57.0)
Separat	18352/104697 (17.5)	4535/24706 (18.4)	6721/40187 (16.7)	3670/21042 (17.4)	3426/18762 (18.3)
Single	17900/104697 (17.1)	5175/24706 (21.0)	5781/40187 (14.4)	3813/21042 (18.1)	3131/18762 (16.7)
Widowed	11339/104697 (10.8)	2234/24706 (9.0)	5112/40187 (12.7)	2477/21042 (11.8)	1516/18762 (8.1)
Country of birth, n/N (%)					
Europe except Sweden	11367/104697 (10.9)	2897/24706 (11.7)	4323/40187 (10.8)	2202/21042 (10.5)	1945/18762 (10.4)
Rest of world	8804/104697 (8.4)	1279/24706 (5.2)	2397/40187 (6.0)	2157/21042 (10.3)	2971/18762 (15.8)
Sweden	84526/104697 (80.7)	20,530 /24706 (83.1)	33467/40187 (83.3)	16683/21042 (79.3)	13846/18762 (73.8)
Education, n/N (%)					
College level	45875/102992 (44.5)	11,178/24300 (46.0)	17124/39569 (43.3)	9332/20686 (45.1)	8241/18437 (44.7)
Elementary school	38725/102992 (37.6)	9384/24300 (38.6)	15796/39569 (39.9)	7592/20686 (36.7)	5953/18437 (32.3)
Upper secondary school	18392/102992 (17.9)	3738/24300 (15.4)	6649/39569 (16.8)	3762/20686 (18.2)	4243/18437 (23.1)
Waist, cm (SD)	104.5 (13.5)	118.4 (11.2)	103.1 (8.9)	100.1 (13.8)	93.9 (9.0)
BMI, kg/m^2^ (SD)	30.3 (5.4)	35.0 (5.3)	29.8 (4.2)	29.1 (5.2)	26.9 (3.7)
HbA1c, mmol/mol (SD)	53.3 (14.7)	61.9 (18.0)	51.2 (12.7)	52.3 (13.9)	47.4 (8.8)
eGDR, mg/kg/min (SD)	5.6 (2.2)	2.9 (1.0)	5.0 (0.6)	6.9 (0.6)	9.1 (0.8)
SBP, mmHg (SD)	137.4 (17.1)	140.3 (17.3)	139.2 (17.0)	136.0 (16.7)	131.1 (15.7)
DBP, mmHg (SD)	78.8 (9.9)	81.0 (10.4)	78.8 (10.0)	78.1 (9.5)	76.8 (9.0)
Total cholesterol, mmol/L (SD)	5.1 (1.1)	5.0 (1.1)	5.0 (1.1)	5.2 (1.1)	5.2 (1.1)
LDL cholesterol, mmol/L (SD)	2.9 (1.0)	2.9 (1.0)	2.9 (1.0)	3.0 (1.0)	3.1 (1.0)
HDL cholesterol, mmol/L (SD)	1.3 (0.4)	1.2 (0.3)	1.3 (0.4)	1.3 (0.4)	1.4 (0.4)
Triglycerides, mmol/L (SD)	1.9 (1.2)	2.3 (1.4)	1.9 (1.1)	1.9 (1.2)	1.6 (1.0)
Creatinine, µmol/L (SD)	76.8 (24.4)	78.9 (28.0)	78.9 (24.8)	74.2 (22.7)	72.4 (18.5)
eGFR, mL/min/1.73 m^2^ (SD)	84.5 (23.9)	85.2 (24.9)	81.4 (23.3)	86.2 (24.5)	88.6 (22.3)
Diabetes treatment, n/N (%)					
Diet only	40763/104697 (38.9)	6110/24706 (24.7)	16092/40187 (40.0)	9303/21042 (44.2)	9258/18762 (49.3)
Insulin	6662/104697 (6.4)	1725/24706 (7.0)	2307/40187 (5.7)	1248/21042 (5.9)	1382/18762 (7.4)
Tablets	48546/104697 (46.4)	13212/24706 (53.5)	18721/40187 (46.6)	9305/21042 (44.2)	7308/18762 (39.0)
Tablets and insulin	8726/104697 (8.3)	3659/24706 (14.8)	3067/40187 (7.6)	1186/21042 (5.6)	814/18762 (4.3)
Anti hypertensive treatment, n/N (%)	70101/104697 (67.0)	24193/24706 (97.9)	37072/40187 (92.3)	8629/21042 (41.0)	207/18762 (1.1)
Lipid lowering treatment, n/N (%)	47144/102086 (46.2)	13289/23932 (55.5)	21337/39106 (54.6)	7520/20587 (36.5)	4998/18461 (27.1)
Microalbuminurea, n/N (%)	9415/69691 (13.5)	3037/15543 (19.5)	3781/26785 (14.1)	1571/14158 (11.1)	1026/13205 (7.8)
Macroalbuminurea, n/N (%)	4759/79042 (6.0)	1712/18058 (9.5)	1929/30447 (6.3)	689/15923 (4.3)	429/14614 (2.9)
Physical Activity, times/week, n/N (%)					
1–2	19205/94161 (20.4)	4937/22029 (22.4)	7469/36215 (20.6)	3778/18990 (19.9)	3021/16927 (17.9)
3–5	2221094161 (23.6)	4250/22029 (19.3)	8746/36215 (24.2)	4641/18990 (24.4)	4573/16927 (27.0)
< 1	1136494161 (12.1)	3758/22029 (17.1)	4109/36215 (11.4)	2087/18990 (11.0)	1410/16927 (8.3)
> 5	3022894161 (32.1)	4977/22029 (22.6)	12009/36215 (33.2)	6503/18990 (34.2%)	6739/16927 (39.8)
Never	1115494161 (11.9)	4107 /22029 (18.6)	3882/36215 (10.7)	1981/18990 (10.4)	1184/16927 (7.0)
History of comorbidities, n/N (%)					
Cardiovascular disease	8135/104697 (7.8)	2561/24706 (10.4)	4232/40187 (10.5)	1039/21042 (4.9)	303/18762 (1.6)
Coronary heart disease	15161/104697 (14.5)	4596/24706 (18.6)	7780/40187 (19.4)	2037/21042 (9.7)	748/18762 (4.0)
Acute myocardial infarction	8135/104697 (7.8)	2561/24706 (10.4)	4232/40187 (10.5)	1039/21042 (4.9)	303/18762 (1.6)
Atrial fibrillation	5415/104697 (5.2)	1734/24706 (7.0)	2554/40187 (6.4)	827/21042 (3.9)	300/18762 (1.6)
Heart failure	4221/104697 (4.0)	1650/24706 (6.7)	1876/40187 (4.7)	526/21042 (2.5)	169/18762 (0.9)
Hyperglycaemia	759/104697 (0.7)	208/24706 (0.8)	260/40187 (0.7)	157/21042 (0.8)	134/18762 (0.7)
Amputation	150/104697 (0.1)	54/24706 (0.2)	60/40187 (0.2)	21/21042 (0.1)	15/18762 (0.1)
Psychiatric disorder	3277/104697 (3.1)	819/24706 (3.3)	1017/40187 (2.5)	777/21042 (3.7)	664/18762 (3.5)
End-stage renal failure	107/104697 (0.1)	27/24706 (0.1)	43/40187 (0.1)	27/21042 (0.13)	10/18762 (0.1)
Cancer	6826/104697 (6.5)	1469/24706 (6.0)	2870/40187 (7.1)	1346/21042 (6.4)	1141/18762 (6.1)
Gastric bypass operation	105/104697 (0.1)	32/24706 (0.1)	38/40187 (0.1)	20/21042 (0.1)	15/18762 (0.1)

*SD* standard deviation

### Association between eGDR and stroke

**Fig. 1:**
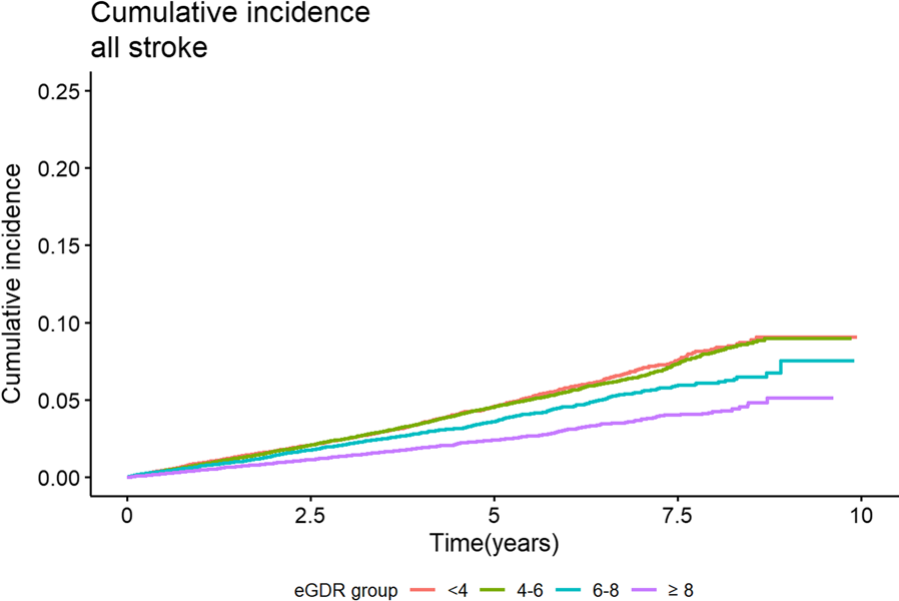
Cumulative incidence of stroke in 104 697 individuals with type 2 diabetes (T2D) separated by the four categories of estimated glucose disposal rate (eGDR)

**Fig. 2:**
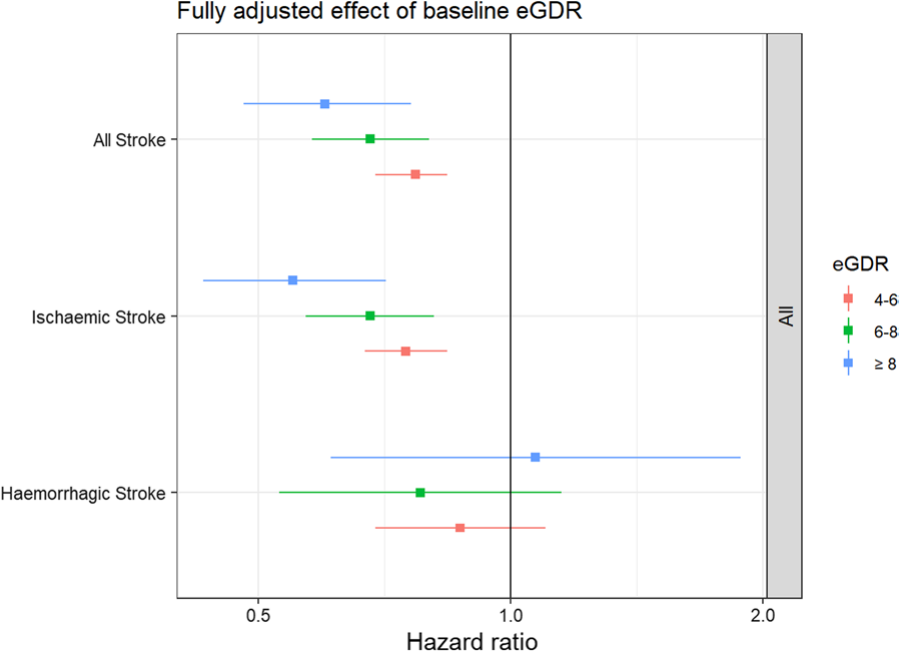
Outcome of stroke divided into all stroke (ischaemic and haemorrhagic stroke), ischaemic and haemorrhagic strokes in 104 697 people with type 2 diabetes in Sweden, from 2004 to 2016, according to estimated glucose disposal rate (eGDR). Reference eGDR < 4 mg/kg/min

Event rates for stroke, i.e., all stroke (ischaemic stroke and haemorrhagic stroke), ischaemic stroke and haemorrhagic stroke, are shown in Table 2. The Kaplan–Meier estimated curves of a first stroke according to the eGDR categories are shown in Fig. 1. During a median follow-up time of 5.6 years, in total 4201 individuals with T2D had a stroke. The crude incidence rates, age-adjusted, and multivariable-adjusted risk of stroke among individuals with T2D, as categorised into different eGDR groups, are shown in Table 2. After multiple adjustments the incidence of all stroke and ischaemic stroke decreased in groups with increasing eGDR (Table 2; Fig. 2). However, for haemorrhagic stroke a non-significant difference was observed between the lowest eGDR compared to the other categories of eGDR (Table 2; Fig. 2).

**Table 2:** Event rates and relative risks for stroke, ischaemic stroke and haemorrhagic stroke, respectively, in 104 697 people with type 2 diabetes, stratified into four groups, depending on estimated glucose disposal rate (eGDR)

Variable	eGDR (mg/kg/min)	Events/person-years	Rate per 1000 person-years (95% CI)	Crude hazard ratio (95% CI)	Age- and sex-adjusted hazard ratio (95% CI)	Multivariable adjusted^*^ hazard ratio (95% CI)
All Stroke	< 4	1106/114389	9.7 (9.1–10.3)	1.00	1.00	1.00
	4–6	1847/194940	9.5 (9.0–9.9)	0.97 (0.90–1.05)	0.78 (0.73–0.84)	0.77 (0.69–0.84)
	6–8	771/100891	7.6 (7.1–8.2)	0.79 (0.72–0.86)	0.73 (0.67–0.81)	0.68 (0.58–0.80)
	> 8	477/93028	5.1 (4.7–5.6)	0.52 (0.47–0.58)	0.59 (0.53–0.65)	0.60 (0.48–0.76)
Ischaemic stroke	< 4	912/114712	8.0 (7.4–8.5)	1.00	1.00	1.00
	4–6	1518/195541	7.8 (7.4–8.2)	0.97 (0.90–1.05)	0.78 (0.71–0.84)	0.75 (0.67–0.84)
	6–8	657/101133	6.5 (6.0–7.0)	0.81 (0.73–0.90)	0.75 (0.68–0.83)	0.68 (0.57–0.81)
	> 8	389/93197	4.2 (3.8–4.6)	0.52 (0.46–0.58)	0.58 (0.51–0.65)	0.55 (0.43–0.71)
Haemorrhagic stroke	< 4	182/116550	1.6 (1.3–1.8)	1.00	1.00	1.00
	4–6	319/198339	1.6 (1.4–1.8)	1.02 (0.85–1.22)	0.85 (0.71–1.03)	0.87 (0.69–1.10)
	6–8	114/102320	1.1 (0.9–1.3)	0.71 (0.56–0.89)	0.72 (0.57–0.91)	0.78 (0.53–1.15)
	> 8	93/93911	1.0 (0.8–1.2)	0.62 (0.48–0.80)	0.73 (0.57–0.93)	1.07 (0.61–1.88)

^*^Multivariable adjusted (see text statistics) not adjusted for variables including in the eGDR formula, i.e. HbA1c, waist circumference and blood pressure

We also analyzed the potentially non-linear association between eGDR levels and stroke risk with restricted cubic splines in a sex and age adjusted Cox regression model. As demonstrated in the (Additional file 1: Figure S3 there was a linear decrease in the risk of ischaemic stroke starting from eGDR 2 mg/kg/min.

### eGDR and risk of stroke stratified by insulin treatment or no insulin treatment

In the cohort 6.4 and 8.3% were treated with insulin only and tablets and insulin, respectively. In the full model after adjusting for insulin treatment no interaction was found for the risk of all stroke p = 0.86, ischaemic stroke p = 0.78, or haemorrhagic stroke p = 0.81, respectively (Additional file 1: Figure S4). Estimates for haemorrhagic stroke were however opposite compared to estimates for all stroke and ischaemic stroke in individuals treated with insulin, although not statistically significantly different from the lowest eGDR category HRs (95% CI): 1.19 (0.63–2.22), 1.63 (0.46–5.8) and 2.67 (0.45–15.74) (Additional file 1: Figure S4).

### Explained variance of the variables for stroke in the eGDR formula

The estimated explained relative risk (R^2^ ± SD) for each variable in the eGDR formula for the risk of stroke was highest for hypertension (0.045 ± 0.0024), followed by HbA1c (0.013 ± 0.0014), and waist (0.006 ± 0.0009) (Additional file 1: Table S3).

### Association between eGDR and mortality after stroke

The event rates for all-cause mortality and cardiovascular mortality are shown in Table 3. During a mean follow up time of 5.6 years in total 3.1% (3232 of 104 697) patients died. According to eGDR categories < 4, 4–6, 6–8 and ≥ 8 mg/kg/min, death occurred, relative to a stroke in 783 (36.6%), 1563 (41.9%), 633 (43.5%) and 253 (37.5%). After multiple adjustments all-cause mortality and cardiovascular mortality rate, after stroke, was lower in individuals categorised in higher eGDR groups (Table 3). HRs (95% CI) for individuals in the highest eGDR category were for all-cause mortality: 0.68 (0.53–0.89) and for cardiovascular mortality 0.65 (0.45–0.93), respectively, compared to individuals in the lowest eGDR category (Table 3).

**Table 3:** Event rates and relative risks for post stroke mortality and cardiovascular mortality in 104 697 patients with type 2 diabetes, stratified in four groups, by estimated glucose disposal rate (eGDR)

Variable	eGDR (mg/kg/min)	Events/person-years	Rate per 1000 person-years (95% CI)	Crude hazard Ratio (95% CI)	Age and sex-adjusted hazard ratio (95% CI)	Multivariable adjusted hazard ratio^*^ (95% CI)
Mortality	< 4	783/5798	135.1 (125.8–144.9)	1.00	1.00	1.00
	4–6	1563/9601	162.8 (154.8–171.1)	1.19 (1.09–1.30)	0.91 (0.83–0.99)	0.82 (0.70–0.94)
	6–8	633/3727	169.8 (156.9–183.6)	1.24 (1.12–1.38)	0.92 (0.83–1.03)	0.75 (0.64–0.88)
	> 8	253/1801	130.5 (114.3–148.3)	0.99 (0.86–1.14)	0.82 (0.70–0.94)	0.68 (0.53–0.89)
Cardiovascular mortality	< 4	421/5798	72.6 (65.8–79.9)	1.00	1.00	1.00
	4–6	851/9601	88.6 (82.8–94.8)	1.20 (1.06–1.34)	0.91 (0.81–1.03)	0.82 (0.70–0.95)
	6–8	344/3727	92.3 (82.8–102.6)	1.24 (1.07–1.43)	0.91 (0.78–1.05)	0.75 (0.60–0.93)
	> 8	122/1801	67.7 (56.3–80.9)	0.97 (0.80–1.19)	0.80 (0.66–0.98)	0.65 (0.45–0.93)

^*^Multivariable adjusted (see text statistics) not adjusted for variables including in the eGDR formula, i.e. HbA1c, waist circumference and blood pressure

#### eGDR calculated using body mass index (eGDR_BMI_)

We also calculated eGDR using BMI instead of waist circumferences, i.e., eGDR_BMI_ (see text, Additional file 1) in which 205 482 individuals with T2D (Additional file 1: Table S4) were included. The correlation between clamp and eGDR_BMI_ was *r* = 0.69 (Additional file 1: Table S1; Figure S1 bottom). All analyses were repeated, and the results are shown in (Additional file 1: Tables S5 and S6 and Figures S5 and S6, respectively. The results were very similar to the results from the main analyses using eGDR from waist circumference. After multiple adjustment the risk of a stroke decreased with increasing eGDR_BMI_ HRs (95% CI): 0.72 (0.67–0.76), 0.56 (0.52–0.61) and 0.39 (0.35–0.44), compared with the reference eGDR_BMI_ < 4 (Additional file 1: Table S5). There was the same increased risk of mortality after a stroke by analysing eGDR_BMI_ (Additional file 1: Table S6). The estimated explained relative risk for stroke (R^2^ ± SD) was higher for BMI (0.021 ± 0.00172) compared to waist (0.006 ± 0.0009) (Additional file 1: Table S3).

## Discussion

This nationwide study, including individuals with T2D, shows that higher eGDR (decreased insulin resistance) was associated with lower risk of stroke and death. The association was independent of clinical characteristics and other identified risk factors for stroke and mortality.

The explained attributable relative risk of a stroke, for each factor in the eGDR formula, was highest for hypertension followed by BMI, HbA1c, and waist circumference, all well known risk factors for stroke [19]. Other known stroke risk factors such as: atrial fibrillation, heart failure, smoking, macrovascular complications, and socioeconomic factors, could also have contributed to the higher risk of stroke observed in the present study [5, 20]. After adjustment for these and a wealth of other risk factors for stroke there was still a monotonic lower risk of stroke and death in the higher eGDR categories compared to the lowest eGDR category, suggesting eGDR as an important risk marker for stroke in individuals with T2D.

Since hypertension is one of the strongest risk factors for stroke, both in people with or without diabetes [19, 21] our findings are not unique and not unexpected. This was also reflected by the attributable risk of the variables in the eGDR formula in which hypertension was most important. In a recent meta-analysis it was shown that a 10 mmHg decrease in systolic blood pressure is associated with a significant decreased risk of stroke people with diabetes [22]. This was also demonstrated in the recent published ONTARGET study whereas individuals with diabetes and cardiovascular risk factors showed a reduction in stroke with a systolic blood pressure up to ≤ 115 mmHg [23]. These studies simply demonstrate the importance to treat hypertension avoiding a stroke. In the INTERSTROKE case–control study, which was launched to investigate the potentially modifiable risk factors associated with stroke, ten strong risk factors for stroke were identified and collectively associated with 90% of patients’ attributable risk [19]. Hypertension had a greater association with haemorrhagic stroke, than with ischaemic stroke, whereas smoking, diabetes, apolipoproteins and cardiovascular disease were more often associated with ischaemic stroke [19]. In the present study, the proportion of stroke events were almost 8 to 9-folded higher for ischaemic stroke, then for haemorrhagic stroke, making the interpretation for the association between risk factors and the risk of haemorrhagic stroke less reliable (less events) compared to ischaemic stroke. However, by using BMI instead of waist circumference in the eGDR formula the number of patients and events were doubled demonstrating the same monotonic pattern for haemorrhagic stroke as for ischaemic stroke.

It was recently shown from the NDR that T2D individuals who had five predefined cardiovascular risk factors, i.e., HbA1c levels, LDL-cholesterol levels, albuminuria, smoking and elevated blood pressure within the clinical target range were not at a higher risk of stroke as for the general population [24]. By using ancillary analyses the strength of the association was estimated among a wealth of risk factors and the risk of cardiovascular outcome, including stroke and mortality. In that study the strongest association for stroke was observed in T2D people with poor glycaemic control, followed by elevated systolic blood pressure, longer duration of diabetes, physical inactivity and atrial fibrillation. Recently, our group confirmed the above results demonstrating a robust association between poor glycaemic control and risk of stroke in individuals with T2D [4]. In the present study, individuals in the lowest eGDR category had a mean HbA1c level of 62 mmol/mol compared to 47 mmol/mol in individuals in the highest eGDR category; since hyperglycaemia is one modifiable risk factor for stroke achievement of good glycaemic control should be strived for to minimise this complication [4].

Obesity has been shown to increase the risk of multiple disease conditions including stroke [25]. In contrast, a survival advantage among individuals with higher BMI has also been suggested for stroke, i.e., the so-called obesity paradox, and whether obesity is an established risk factor for stroke is still debated [26]. In a large observational study among people with type 1 diabetes however no such paradox exists [27]. It has also been reported among overweight and obese T2D individuals a considerably independent increased risk for stroke and total mortality [28]. Increased waist-to-hip ratio was also a strong risk marker in the INTERSTROKE case–control study [19]. Since, waist circumference reflects central obesity and independently is associated with insulin resistance and cardiovascular disease, we used this variable in the main eGDR analysis. After replacement with BMI, instead of waist circumference in the eGDR formula, there was the same robust monotonic decrease in a stroke event and mortality thereof above reference eGDR category, and the estimated explained attributable risk was even higher for BMI compared to waist circumference. Even though an obesity paradox may exist it has not been proven in large observational studies (like the present), whereas both waist circumference and BMI associate to the risk of stroke.

Although our data from euglycaemic hyperinsulinaemic clamps, to some extent correlated to the eGDR formula used for individuals with type 1 diabetes [29], we cannot tell whether insulin resistance contributes for the excess risk of stroke or mortality observed in the present study. The impact of insulin resistance on cardiovascular disease is not easy to determine since it is clustering with several other traditional risk factors, e.g., hypertension, obesity, elevated triglycerides, low levels of HDL-cholesterol and hyperglycaemia [10]. Interestingly, it has been evaluated in a mathematical model that preventing insulin resistance would yield as much as 40% prevention of cardiovascular disease in young adults [6]. Even though insulin resistance is suggested the most important single risk factor for cardiovascular disease, its effect is indirect mediated thorough its effect on other variables such as blood pressure, lipidaemia and glycaemia [6]. When we adjusted for variables known to be linked between insulin resistance and cardiovascular complications, eGDR above reference was strongly associated with a reduced risk of stroke and death thereof in a stepwise manner. Insulin therapy, often needed to combat hyperglycaemia in T2D individuals, has been proven safe in large randomised controlled cardiovascular outcome trials [30, 31]. In contrast, some large observational studies have come to another conclusion, suggesting an association between insulin therapy in people with T2D and cardiovascular disease or premature death [32]. In the present study there was no interaction between insulin therapy and stroke. The estimates for haemorrhagic stroke in the insulin treated individuals did however not follow the pattern as for ischaemic stroke, which simply can be explained by the very small number of events in this group.

The strength of this study was its unique nationwide coverage with a large cohort of T2D individuals; and a long follow up-time with accurate and register data with high external and internal validity. However, there are some limitations; eGDR is a measure of insulin resistance developed for individuals with type 1 diabetes, although the correlation between euglycaemic hyperinsulinaemic clamp and eGDR were good (however only tested for men) we cannot conclude that eGDR can be replaced with the gold standard clamp technique. Another limitation is that we lack information of medical therapy for secondary prevention, nor did we in this study have any data on the use of antidiabetic medication such as incretins and sodium glucose transporter-2 inhibitors which has been proven beneficial on cardiovascular mortality, and may have different impact on the risk of stroke. As in any observational study, we cannot rule out that residual confounding factors affected our findings.

In conclusion we found that in individuals with T2D a low eGDR, a simple measure of insulin resistance, was associated with an increased risk of a stroke and mortality. Consequently, insulin resistance seems to increase the risk for stroke in type 2 diabetes, and eGDR may be used as a risk marker for stroke and death.

## Supplementary Information

**Additional file 1: Table S1**. Clinical characteristics of 24 male patients with type 2 diabetes who underwent a hyperinsulinaemic clamp procedure (CLAMP) and its comparison with estimated glucose disposal rate (eGDR) based on waist (eGDRwaist) and BMI (eGDRBMI), respectively. **Table S2**. 9th and 10th revision of international Classification of Diseases Codes (ICD-codes). **Table S3**. Hellers R2 (a measure of explained variance) calculated for HbA1c, waist, BMI and hypertension used as single main effects predictors in the Cox regression models (2). **Table S4**. Baseline characteristics of 205 482 patients with type 2 diabetes mellitus categorised in 4 groups of estimated glucose disposal rate (eGDR) based on BMI (eGDRBMI). **Table S5**. Event rates and relative risks for stroke, ischaemic stroke and haemorrhagic stroke, respectively, in 205 482 people with type 2 diabetes, stratified into four groups, depending on estimated glucose disposal rate (eGDR). **Table S6**. Event rates and relative risks unadjusted and adjusted for all-cause mortality and cardiovascular mortality in 205 482 people with type 2 diabetes, stratified into four groups, depending on estimated glucose disposal rate (eGDRBMI). **Figure S1**. Spearman association curves (r-value) between M-values for the hyperinsulinemic clamp (X-axis) and estimated glucose disposal rate (y-axis) based on waist (eGDRwaist) and BMI (eGDRBMI) top and bottom, respectively. **Figure S2**. Flowchart for the studied group. **Figure S3**. Adjusted hazard ratio (solid line) and 95% confidence intervals (dashed lines) for the association between baseline eGDR and stroke. The baseline eGDR level was modelled with restricted cubic splines in a Cox regression model adjusted for sex and age. **Figure S4. **Fully adjusted hazard ratios for stroke; on insulin treatment and not on insulin treatment, divided into all stroke (ischaemic and haemorrhagic stroke), ischaemic and haemorrhagic stroke, respectively in 104 697 individuals with type 2 diabetes according to eGDR (Reference eGDR <4). **Figure S5. **Cumulative incidence of stroke in in 205 482 patients with type 2 diabetes, divided into different groups depending on eGDRBMI. **Figure S6**. Hazard ratios for a first stroke, fully adjusted, divided into all stroke (ischaemic and haemorrhagic stroke), ischaemic and haemorrhagic stroke in 205 482 individuals with type 2 diabetes according to eGDRBMI (Reference eGDRBMI <4).

## Data Availability

The datasets analysed during the current study are available from the corresponding author on reasonable request.

## Abbreviations

BMI: Body mass index
CI: Confidence interval
CHD: Coronary heart disease
DPP4i: Dipeptidyl peptidase 4 inhibitors
eGDR: Estimated glucose disposal rate
GLP-1: Glucagon-like peptide-1
HbA1c: Glycosylated haemoglobin A1c
HR: Hazard ratio
ICD: International Classification of Disease
IPR: In patient registry
IQR: Interquartile range
LISA: Longitudinal integration database for health insurance and job market studies register
MACE: Major adverse cardiac event
MI: Myocardial infarction
NDR: National diabetes register
NPR: Swedish national patient register
OR: Odds rate
SD: Standard deviation
SLGT2i: Sodium glucose transporter-2 inhibitors
T2D: Type 2 diabetes

## References

[1] Shaw JE, Sicree RA, Zimmet PZ (2010). Global estimates of the prevalence of diabetes for 2010 and 2030. Diabetes Res Clin Pract.

[2] Cederholm J, Eeg-Olofsson K, Eliasson B, Zethelius B, Nilsson PM, Gudbjörnsdottir S (2008). Risk prediction of cardiovascular disease in type 2 diabetes: a risk equation from the Swedish National Diabetes Register. Diabetes Care.

[3] Collaboration APCS (2003). The effects of diabetes on the risks of major cardiovascular diseases and death in the Asia-Pacific Region. Diabetes Care.

[4] Zabala A, Darsalia V, Holzmann MJ, Franzén S, Svensson A-M, Eliasson B (2020). Risk of first stroke in people with type 2 diabetes and its relation to glycaemic control: a nationwide observational study. Diabetes Obes Metab.

[5] Ormazabal V, Nair S, Elfeky O, Aguayo C, Salomon C, Zuñiga FA (2018). Association between insulin resistance and the development of cardiovascular disease. Cardiovasc Diabetol.

[6] Eddy D, Schlessinger L, Kahn R, Peskin B, Schiebinger R (2009). Relationship of insulin resistance and related metabolic variables to coronary artery disease: a mathematical analysis. Diabetes Care.

[7] DeFronzo RA, Tobin JD, Andres R (1979). Glucose clamp technique: a method for quantifying insulin secretion and resistance. Am J Physiol.

[8] Williams KV, Erbey JR, Becker D, Arslanian S, Orchard TJ (2000). Can clinical factors estimate insulin resistance in type 1 diabetes?. Diabetes.

[9] Nyström T, Holzmann MJ, Eliasson B, Svensson A-M, Sartipy U (2018). Estimated glucose disposal rate predicts mortality in adults with type 1 diabetes. Diabetes Obes Metab.

[10] Nyström T, Holzmann MJ, Eliasson B, Svensson A-M, Kuhl J, Sartipy U (2017). Estimated glucose disposal rate and long-term survival in type 2 diabetes after coronary artery bypass grafting. Heart Vessels.

[11] Fonville S, Zandbergen AAM, Koudstaal PJ, den Hertog HM (2014). Prediabetes in patients with stroke or transient ischemic attack: prevalence, risk and clinical management. Cerebrovasc Dis Basel Switz.

[12] Kernan WN, Inzucchi SE, Viscoli CM, Brass LM, Bravata DM, Horwitz RI (2002). Insulin resistance and risk for stroke. Neurology.

[13] Eliasson B, Gudbjörnsdottir S (2014). Diabetes care—improvement through measurement. Diabetes Res Clin Pract.

[14] Cederholm J, Gudbjörnsdottir S, Eliasson B, Zethelius B, Eeg-Olofsson K, Nilsson PM (2012). Blood pressure and risk of cardiovascular diseases in type 2 diabetes: further findings from the Swedish National Diabetes Register (NDR-BP II). J Hypertens.

[15] Appelros P, Terént A (2011). Validation of the Swedish inpatient and cause-of-death registers in the context of stroke. Acta Neurol Scand.

[16] Ludvigsson JF, Svedberg P, Olén O, Bruze G, Neovius M (2019). The longitudinal integrated database for health insurance and labour market studies (LISA) and its use in medical research. Eur J Epidemiol.

[17] Hoelzel W, Weykamp C, Jeppsson J-O, Miedema K, Barr JR, Goodall I (2004). IFCC reference system for measurement of hemoglobin A1c in human blood and the national standardization schemes in the United States, Japan, and Sweden: a method-comparison study. Clin Chem.

[18] Heller G (2012). A measure of explained risk in the proportional hazards model. Biostat Oxf Engl.

[19] O’Donnell MJ, Xavier D, Liu L, Zhang H, Chin SL, Rao-Melacini P (2010). Risk factors for ischaemic and intracerebral haemorrhagic stroke in 22 countries (the INTERSTROKE study): a case-control study. The Lancet.

[20] Lee Y, Cha SJ, Park J-H, Shin J-H, Lim Y-H, Park H-C (2020). Association between insulin resistance and risk of atrial fibrillation in non-diabetics. Eur J Prev Cardiol.

[21] Hu G, Sarti C, Jousilahti P, Peltonen M, Qiao Q, Antikainen R (2005). The impact of history of hypertension and type 2 diabetes at baseline on the incidence of stroke and stroke mortality. Stroke.

[22] Xie X-X, Liu P, Wan F-Y, Lin S-G, Zhong W-L, Yuan Z-K (2016). Blood pressure lowering and stroke events in type 2 diabetes: a network meta-analysis of randomized controlled trials. Int J Cardiol.

[23] Redon J, Mancia G, Sleight P, Schumacher H, Gao P, Pogue J (2012). Safety and efficacy of low blood pressures among patients with diabetes: subgroup analyses from the ONTARGET (ONgoing Telmisartan Alone and in combination with Ramipril Global Endpoint Trial). J Am Coll Cardiol.

[24] Rawshani A, Rawshani A, Franzén S, Sattar N, Eliasson B, Svensson A-M (2018). Risk factors, mortality, and cardiovascular outcomes in patients with type 2 diabetes. N Engl J Med.

[25] Upadhyay J, Farr O, Perakakis N, Ghaly W, Mantzoros C (2018). Obesity as a disease. Med Clin North Am.

[26] Oesch L, Tatlisumak T, Arnold M, Sarikaya H (2017). Obesity paradox in stroke—Myth or reality? A systematic review. PLoS ONE.

[27] Edqvist J, Rawshani A, Adiels M, Björck L, Lind M, Svensson A-M (2019). BMI, mortality, and cardiovascular outcomes in type 1 diabetes: findings against an obesity paradox. Diabetes Care.

[28] Eeg-Olofsson K, Cederholm J, Nilsson PM, Zethelius B, Nunez L, Gudbjörnsdóttir S (2009). Risk of cardiovascular disease and mortality in overweight and obese patients with type 2 diabetes: an observational study in 13,087 patients. Diabetologia.

[29] Epstein EJ, Osman JL, Cohen HW, Rajpathak SN, Lewis O, Crandall JP. Use of the estimated glucose disposal rate (eGDR) as a Measure of Insulin resistance in an urban multiethnic population with type 1 diabetes. Diabetes Care. 2013 Apr 12 [cited 2021 Jun 2]; https://care.diabetesjournals.org/content/early/2013/04/12/dc12-1693 10.2337/dc12-1693 PMC3714518 23596179

[30] UK Prospective Diabetes Study (UKPDS) Group (1998). Intensive blood-glucose control with sulphonylureas or insulin compared with conventional treatment and risk of complications in patients with type 2 diabetes (UKPDS 33). Lancet Lond Engl.

[31] ORIGIN Trial Investigators, Gerstein HC, Bosch J, Dagenais GR, Díaz R, Jung H, et al. Basal insulin and cardiovascular and other outcomes in dysglycemia. N Engl J Med. 2012;367(4):319–28. 10.1056/NEJMoa1203858 22686416

[32] Currie CJ, Peters JR, Tynan A, Evans M, Heine RJ, Bracco OL (2010). Survival as a function of HbA(1c) in people with type 2 diabetes: a retrospective cohort study. Lancet Lond Engl.

