# Supplementary material for: Estimated glucose disposal rate and risk of stroke and mortality in type 2 diabetes: a nationwide cohort study
Source: Cardiovasc Diabetol. 2021 Oct 6;20:202. doi: 10.1186/s12933-021-01394-4 (PMC8495918; doi:10.1186/s12933-021-01394-4)
Supplement: Supplementary file 1 — Additional file 1: Table S1. Clinical characteristics of 24 male patients with type 2 diabetes who underwent a hyperinsulinaemic clamp procedure (CLAMP) and its comparison with estimated glucose disposal rate (eGDR) based on waist (eGDRwaist) and BMI (eGDRBMI), respectively. Table S2. 9th and 10th revision of international Classification of Diseases Codes (ICD-codes). Table S3. Hellers R2 (a measure of explained variance) calculated for HbA1c, waist, BMI and hypertension used as single main effects predictors in the Cox regression models (2). Table S4. Baseline characteristics of 205 482 patients with type 2 diabetes mellitus categorised in 4 groups of estimated glucose disposal rate (eGDR) based on BMI (eGDRBMI). Table S5. Event rates and relative risks for stroke, ischaemic stroke and haemorrhagic stroke, respectively, in 205 482 people with type 2 diabetes, stratified into four groups, depending on estimated glucose disposal rate (eGDR). Table S6. Event rates and relative risks unadjusted and adjusted for all-cause mortality and cardiovascular mortality in 205 482 people with type 2 diabetes, stratified into four groups, depending on estimated glucose disposal rate (eGDRBMI). Figure S1. Spearman association curves (r-value) between M-values for the hyperinsulinemic clamp (X-axis) and estimated glucose disposal rate (y-axis) based on waist (eGDRwaist) and BMI (eGDRBMI) top and bottom, respectively. Figure S2. Flowchart for the studied group. Figure S3. Adjusted hazard ratio (solid line) and 95% confidence intervals (dashed lines) for the association between baseline eGDR and stroke. The baseline eGDR level was modelled with restricted cubic splines in a Cox regression model adjusted for sex and age. Figure S4. Fully adjusted hazard ratios for stroke; on insulin treatment and not on insulin treatment, divided into all stroke (ischaemic and haemorrhagic stroke), ischaemic and haemorrhagic stroke, respectively in 104 697 individuals with type 2 diabetes according to [file 12933_2021_1394_MOESM1_ESM.docx]

**Supplementary Material**

**Estimated glucose disposal rate and risk of first stroke and mortality in type 2 diabetes: a nationwide cohort study**

Alexander Zabala^1^, Vladimer Darsalia^1^, Marcus Lind^2,3^, Ann-Marie Svensson^4^, Stefan Franzén^4^, Björn Eliasson^2^, Cesare Patrone^1^ Magnus Jonsson^5,6^, Thomas Nyström^1^

^1^Department of Clinical Science and Education, Karolinska Institutet, Södersjukhuset, Stockholm, Sweden

^2^Institute of Medicine, University of Gothenburg, Gothenburg, Sweden

^3^Department of Medicine, NU Hospital Group, Uddevalla, Sweden.

^4^Centre of Registers in Region Västra Götaland, Sweden

^5^Department of Molecular Medicine and Surgery, Karolinska Institutet, Stockholm, Sweden.

^6^Department of Vascular Surgery, Karolinska University Hospital, Stockholm, Sweden

## eGDR based on body mass index

eGDR based on body mass index was calculated according to the following formula (personal communication from Kathy Williams) (1).

**eGDR_BMI_** = 19.02 − (0.22 * BMI) − (3.26 * HT) − (0.61 * HbA1c)

*BMI = body mass index (kg/m^2^), HT = hypertension (yes = 1/no = 0), and HbA1c = HbA1c (%).*

**Table S1.** Clinical characteristics of 24 male patients with type 2 diabetes who underwent a hyperinsulinaemic clamp procedure (CLAMP) and its comparison with estimated glucose disposal rate (eGDR) based on waist (eGDR_waist_) and BMI (eGDR_BMI_), respectively.

| **Patients** | **Yrs** | **Hypertension**  **Yes/No** | **HbA_1c_**  **%** | **Waist**  **cm** | **BMI**  **kg/m^2^** | **eGDR_Waist_**  **mg/kg/min** | **eGDR_BMI_**  **mg/kg/min** | **CLAMP**  **mg/kg/min** |
| --- | --- | --- | --- | --- | --- | --- | --- | --- |
| 1 | 66 | Yes | 6.5 | 99 | 24.9 | 5.26 | 6.32 | 3.83 |
| 2 | 57 | Yes | 7.7 | 126 | 36.2 | 2.17 | 3.10 | 1.88 |
| 3 | 60 | Yes | 8.4 | 109.5 | 26.2 | 3.27 | 4.87 | 2.95 |
| 4 | 64 | No | 6.1 | 96 | 25.4 | 9.12 | 9.71 | 5.16 |
| 5 | 60 | Yes | 7.4 | 107 | 30.1 | 4.04 | 4.62 | 3.20 |
| 6 | 50 | Yes | 9.2 | 110 | 23.7 | 2.78 | 4.93 | 2.11 |
| 7 | 55 | Yes | 6.3 | 124 | 33.6 | 3.12 | 4.26 | 3.00 |
| 8 | 50 | Yes | 5.7 | 111 | 31.3 | 4.62 | 5.40 | 4.38 |
| 9 | 55 | Yes | 6.2 | 111 | 29.6 | 4.35 | 5.47 | 4.61 |
| 10 | 56 | Yes | 7.3 | 109 | 30.7 | 3.92 | 4.55 | 1.59 |
| 11 | 56 | Yes | 8.1 | 111 | 30.6 | 3.30 | 4.09 | 4.21 |
| 12 | 60 | Yes | 7.1 | 100 | 26.9 | 4.84 | 5.51 | 2.90 |
| 13 | 46 | Yes | 7.6 | 107 | 30.7 | 3.93 | 4.37 | 2.43 |
| 14 | 69 | Yes | 6.4 | 88 | 25.9 | 6.31 | 6.16 | 5.97 |
| 15 | 70 | Yes | 8.0 | 110 | 28.7 | 3.44 | 4.57 | 3.34 |
| 16 | 60 | No | 9.6 | 112 | 29.8 | 5.79 | 6.61 | 4.54 |
| 17 | 51 | No | 6.1 | 111 | 30.7 | 7.81 | 8.56 | 4.99 |
| 18 | 67 | Yes | 8.0 | 96 | 27.1 | 4.70 | 4.92 | 4.42 |
| 19 | 58 | Yes | 6.9 | 98 | 26.6 | 5.13 | 5.70 | 3.38 |
| 20 | 74 | Yes | 7.2 | 99 | 26.7 | 4.87 | 5.49 | 4.25 |
| 21 | 62 | Yes | 7.2 | 88 | 22.4 | 5.86 | 6.44 | 5.87 |
| 22 | 46 | Yes | 7.9 | 99 | 27.5 | 4.49 | 4.89 | 2.40 |
| 23 | 65 | Yes | 9.2 | 116 | 28.4 | 2.24 | 3.90 | 1.36 |
| 24 | 52 | Yes | 10.2 | 129.5 | 37.4 | 0.48 | 1.31 | 2.02 |

**Table S2.** 9^th^ and 10^th^ revision of international Classification of Diseases Codes (ICD-codes)

| **Diagnosis** | **ICD 9-codes** | | **ICD 10-codes** |
| --- | --- | --- | --- |
| Acute Myocardial Infarction | | 410 | I21 |
| Amputation | |  | Z89.4-7 |
| Atrial Fibrillation | | 427.3 | I48 |
| Cancer | | 140-208 | C00-C97 |
| Coronary Heart Disease | | 410-414 | I20-25 |
| Dementia | |  | F00-F03 |
| Gastric by-pass surgery | |  | JDF10, 11, 50, 51 |
| Heart Failure | | 428 | I50 |
| Psychiatric disorders | |  | F20-29, F30-39 |
| Renal disorder | | V42A, V45B, V56A, V56W | Z94.0, Z49.0-2, Z99.2 |

Table S3. Hellers R^2^ (a measure of explained variance) calculated for HbA1c, waist, BMI and hypertension used as single main effects predictors in the Cox regression models (2)

| Event | Variable | Est | Se |
| --- | --- | --- | --- |
| All stroke | HbA1c | 0.0132598 | 0.0013572 |
|  | Waist | 0.0061227 | 0.0009342 |
|  | BMI | 0.0208077 | 0.0017177 |
|  | Hypertension | 0.0447911 | 0.0023465 |
| **Haemorrhagic** Stroke | HbA1c | 0.0018045 | 0.0012769 |
|  | Waist | 0.0013738 | 0.0011088 |
|  | BMI | 0.0208382 | 0.0042994 |
|  | Hypertension | 0.0412183 | 0.0056206 |
| Ischaemic Stroke | HbA1c | 0.0152809 | 0.0015836 |
|  | Waist | 0.0066114 | 0.0010598 |
|  | BMI | 0.0196034 | 0.0018247 |
|  | Hypertension | 0.0473869 | 0.0026275 |

**Table S4.** Baseline characteristics of 205 482 patients with type 2 diabetes mellitus categorised in 4 groups of estimated glucose

disposal rate (eGDR) based on BMI (eGDR_BMI_)

|  | **All**  **(n=205 482)** | **eGDR_BMI_ <4**  **mg/kg/min (n=36 323)** | **eGDR_BMI_ 4-6**  **mg/kg/min (n=78 817)** | **eGDR_BMI_ 6-8**  **mg/kg/min (n=48 073)** | **eGDR_BMI_ >8**  **mg/kg/min (n=42 269)** |
| --- | --- | --- | --- | --- | --- |
| **Age, yrs (SD)** | 63.2 (11.8) | 62.3 (10.2) | 65.3 (10.7) | 62.0 (12.7) | 59.5 (12.2) |
| **Age at onset, yrs (SD)** | 58.7 (11.8) | 57.6 (10.3) | 60.9 (10.8) | 58.3 (12.5) | 56.1 (12.3) |
| **Diabetes duration, yrs (SD)** | 4.6 (6.0) | 4.7 (6.1) | 4.3 (5.8) | 3.6 (5.4) | 3.5 (5.3) |
| **Women, n/N (%)** | 92763/205482 (45.1) | 18162/36323 (50.0) | 34961/78817 (44.4) | 22333/48073 (46.5) | 19974/42269 (47.3) |
| **Smoking, n/N (%)** | 30258/186153 (16.3) | 4944/32554 (15.2) | 10377/71522 (14.5) | 7624/43597 (17.5) | 7313/38480 (19.0) |
| **Marital status, n/N (%)** |  |  |  |  |  |
| **Married** | 35344/205482 (17.2) | 18020/36323 (49.6) | 43728/78817 (55.5) | 25996/48073 (54.1) | 2047/42269 (4.84) |
| **Separat** | 34340/205482 (16.7) | 6747/36323 (18.6) | 13042/78817 (16.6) | 8052/48073 (16.8) | 7503/42269 (17.8) |
| **Single** | 24259/205482 (11.8) | 7487/36323 (20.6) | 11386/78817 (14.5) | 8018/48073 (16.7) | 7449/42269 (17.6) |
| **Widowed** | 111539/205482 (54.3) | 4069/36323 (11.2) | 10661/78817 (13.5) | 6007/48073 (12.5) | 3522/42269 (8.3) |
| **Country of birth, n/N (%)** |  |  |  |  |  |
| **Europe except Sweden** | 16545/205482 (8.1) | 4818/36323 (13.3) | 8698/78817 (11.0) | 4767/48073 (9.9) | 4013/42269 (9.5) |
| **Rest of world** | 166641/205482 (81.1) | 2192/36323 (6.0) | 4536/78817 (5.8) | 4336/48073 (9.0) | 5481/42269 (13.0) |
| **Sweden** | 22296/205482 (10.9) | 29313/36323 (80.7) | 65583/78817 (83.2) | 38970/48073 (81.1) | 32775/42269 (77.5) |
| **Education, n/N (%)** |  |  |  |  |  |
| **College level** | 80178/201749 (39.7) | 16032/35555 (45.1) | 32574/77419 (42.1) | 20410/47240 (43.2) | 18263/41535 (44.0) |
| **Elementary school** | 34292/201749 (17.0) | 14523/35555 (40.9) | 32907/77419 (42.5) | 18581/47240 (39.3) | 14167/41535 (34.1) |
| **Upper secondary school** | 87279/201749 (43.3) | 5000/35555 (14.1) | 11938/77419 (15.4) | 8249/47240 (17.5) | 9105/41535 (21.9) |
| **BMI, kg/m^2^ (SD)** | 30.2 (5.5) | 35.0 (5.3) | 29.8 (4.2) | 29.1 (5.2) | 26.9 (3.7) |
| **HbA1c, mmol/mol (SD)** | 53.6 (14.8) | 61.9 (18.0) | 51.2 (12.7) | 52.3 (13.9) | 47.4 (8.8) |
| **eGDR, mg/kg/min (SD)** | 5.6 (2.2) | 2.9 (1.0) | 5.0 (0.6) | 6.9 (0.6) | 9.1 (0.8) |
| **SBP, mmHg (SD)** | 138.2 (17.3) | 140.3 (17.3) | 139.2 (17.0) | 136.0 (16.7) | 131.1 (15.7) |
| **DBP, mmHg (SD)** | 78.7 (9.8) | 81.0 (10.4) | 78.8 (10.0) | 78.1 (9.5) | 76.8 (9.0) |
| **Total cholesterol, mmol/L (SD)** | 5.1 (1.1) | 5.0 (1.1) | 5.0 (1.1) | 5.2 (1.1) | 5.2 (1.1) |
| **LDL cholesterol, mmol/L (SD)** | 2.9 (1.0) | 2.9 (1.0) | 2.9 (1.0) | 3.0 (1.0) | 3.1 (1.0) |
| **HDL cholesterol, mmol/L (SD)** | 1.3 (0.4) | 1.2 (0.3) | 1.3 (0.4) | 1.3 (0.4) | 1.4 (0.4) |
| **Triglycerides, mmol/L (SD)** | 1.9 (1.2) | 2.3 (1.4) | 1.9 (1.1) | 1.9 (1.2) | 1.6 (1.0) |
| **Creatinine, µmol/L (SD)** | 77.9 (26.1) | 78.9 (28.0) | 78.9 (24.8) | 74.2 (22.7) | 72.4 (18.5) |
| **eGFR, mL/min/1.73 m^2^ (SD)** | 83.3 (24.1) | 85.2 (24.9) | 81.4 (23.3) | 86.2 (24.5) | 88.6 (22.3) |
| **Diabetes treatment, n/N (%)** |  |  |  |  |  |
| **Diet only** | 77834/205482 (37.9) | 8227/36323 (22.7) | 28500/78817 (36.2) | 21133/48073 (44.0) | 19974/42269 (47.3) |
| **Insulin** | 15619/205482 (7.6) | 2784/36323 (7.7) | 5563/78817 (7.1) | 3393/48073 (7.1) | 3879/42269 (9.2) |
| **Tablets** | 93709/205482 (45.6) | 19199/36323 (52.9) | 37500/78817 (47.6) | 20641/48073 (42.9) | 16369/42269 (38.7) |
| **Tablets and insulin** | 18320/205482 (8.9) | 6113/36323 (16.8) | 7254/78817 (9.2) | 2906/48073 (6.0) | 2047/42269 (4.8) |
| **Anti hypertensive treatment, n/N (%)** | 135619/205482 (66.0) | 35194/36323 (96.9) | 73194/78817 (92.9) | 26908/48073 (56.0) | 323/42269 (0.8) |
| **Lipid lowering treatment, n/N (%)** | 88562/200392 (44.2) | 18348/35100 (52.3) | 40851/76724 (53.2) | 18762/46952 (40.0) | 10601/41616 (25.5) |
| **Microalbuminurea, n/N (%)** | 19188/133940 (14.3) | 4511/22322 (20.2) | 8075/50984 (15.8) | 4101/31452 (13.0) | 2501/29182 (8.6) |
| **Macroalbuminurea, n/N (%)** | 9465/156539 (6.1) | 2561/26738 (9.6) | 4076/60056 (6.8) | 1845/36618 (5.0) | 983/33127 (3.0) |
| **Physical Activity, times/week, n/N (%)** |  |  |  |  |  |
| **1-2** | 31151/146672 (21.2) | 5913/26163 (22.6) | 12608/56941 (22.1) | 6856/34211 (20.0) | 5774/29357 (19.7) |
| **3-5** | 33724/146672 (23.0) | 4646/26163 (17.8) | 13135/56941 (23.1) | 8196/34211 (24.0) | 7747/29357 (26.4) |
| **<1** | 18621/146672 (12.7) | 4973/26163 (19.0) | 7225/56941 (12.7) | 3772/34211 (11.0) | 2651/29357 (9.0) |
| **>5** | 44484/146672 (30.3) | 5082/26163 (19.4) | 16995/56941 (29.9) | 11603/34211 (33.9) | 10804/29357 (36.8) |
| **Never** | 18692/146672 (12.7) | 5549/26163 (21.2) | 6978/56941 (12.3) | 3784/34211 (11.1) | 2381/29357 (8.1) |
| **History of comorbidities, n/N (%)** |  |  |  |  |  |
| **Cardiovascular disease** | 16474/205482 (8.0) | 3479/36323 (9.6) | 8481/78817 (10.8) | 3583/48073 (7.5) | 931/42269 (2.2) |
| **Coronary heart disease** | 30817/205482 (15.0) | 6409/36323 (17.6) | 15773/78817 (20.0) | 6622/48073 (13.8) | 2013/42269 (4.8) |
| **Acute myocardial infarction** | 16474/205482 (8.0) | 3479/36323 (9.6) | 8481/78817 (10.8) | 3583/48073 (7.5) | 931/42269 (2.2) |
| **Atrial fibrillation** | 11317/205482 (5.5) | 2505/36323 (6.9) | 5410/78817 (6.9) | 2540/48073 (5.3) | 862/42269 (2.0) |
| **Heart failure** | 9196/205482 (4.5) | 2563/36323 (7.1) | 4340/78817 (5.5) | 1813/48073 (3.8) | 480/42269 (1.1) |
| **Hyperglycaemia** | 1761/205482 (0.8) | 378/36323 (1.0) | 636/78817 (0.8) | 363/48073 (0.8) | 384/42269 (0.9) |
| **Amputation** | 355/205482 (0.2) | 78/36323 (0.2) | 136/78817 (0.2) | 87/48073 (0.2) | 54/42269 (0.1) |
| **Psychiatric disorder** | 6248/205482 (3.0) | 1231/36323 (3.4) | 1921/78817 (2.4) | 1599/48073 (3.3) | 1497/42269 (3.5) |
| **End-stage renal failure** | 314/205482 (0.2) | 43/36323 (0.1) | 131/78817 (0.2) | 100/48073 (0.2) | 40/42269 (0.1) |
| **Cancer** | 13889/205482 (6.8) | 2257/36323 (6.2) | 5654/78817 (7.2) | 3339/48073 (7.0) | 2639/42269 (6.2) |
| **Gastric bypass operation** | 203/205482 (0.1) | 59/36323 (0.2) | 64/78817 (0.1) | 41/48073 (0.1) | 39/42269 (0.1) |

SD, Standard deviation

**Table S5.** Event rates and relative risks for stroke, ischaemic stroke and haemorrhagic stroke, respectively, in 205 482 people with

type 2 diabetes, stratified into four groups, depending on estimated glucose disposal rate (eGDR)

| **Variable** | **eGDR_BMI_ (mg/kg/min)** | **Events**  **person-years** | **Rate per 1000**  **person-years**  **(95% CI)** | **Crude hazard ratio**  **(95% CI)** | **Age and sex adjusted hazard ratio (95% CI)** | **Multivariable adjusted^*^ hazard ratio (95% CI)** |
| --- | --- | --- | --- | --- | --- | --- |
| **All Stroke** | **<4** | 1797/185156 | 9.7 (9.3-10.2) | 1.00 | 1.00 | 1.00 |
|  | **4-6** | 4598/421543 | 10.9 (10.6-11.2) | 1.05 (1.01-1.09) | 0.81 (0.77-0.84) | 0.72 (0.68-0.76) |
|  | **6-8** | 2398/256339 | 9.4 (9.0-9.7) | 0.88 (0.85-0.92) | 0.73 (0.70-0.76) | 0.58 (0.54-0.63) |
|  | **>8** | 1481/236559 | 6.3 (5.9-6.6) | 0.64 (0.61-0.67) | 0.60 (0.57-0.63) | 0.42 (0.38-0.47) |
| **Ischaemic stroke** | **<4** | 1516/185724 | 8.2 (7.4-8.5) | 1.00 | 1.00 | 1.00 |
|  | **4-6** | 3858/422969 | 9.1 (7.4-8.2) | 0.97 (0.90-1.05) | 0.81 (0.78-0.85) | 0.72 (0.67-0.76) |
|  | **6-8** | 1985/257135 | 7.7 (6.0-7.0) | 0.81 (0.73-0.90) | 0.72 (0.69-0.76) | 0.56 (0.52-0.61) |
|  | **>8** | 1215/237090 | 5.1 (3.8-4.6) | 0.52 (0.46-0.58) | 0.59 (0.56-0.62) | 0.39 (0.35-0.44) |
| **Haemorrhagic stroke** | **<4** | 276/189014 | 1.5 (1.3-1.8) | 1.00 | 1.00 | 1.00 |
|  | **4-6** | 706/431179 | 1.6 (1.4-1.8) | 1.08 (0.98-1.02) | 0.84 (0.76-0.94) | 0.81 (0.70-0.93) |
|  | **6-8** | 391/261099 | 1.5 (0.9-1.3) | 0.97 (0.87-1.09) | 0.81 (0.73-0.91) | 0.81 (0.67-0.98) |
|  | **>8** | 263/239741 | 1.1 (0.8-1.2) | 0.74 (0.66-0.83) | 0.68 (0.61-0.77) | 0.74 (0.56-0.98) |

*Multivariable adjusted (see text statistics) not adjusted for variables including in the eGDR formula, i.e. HbA1c, waist circumference and

blood pressure.

**Table S6.** Event rates and relative risks unadjusted and adjusted for all-cause mortality and cardiovascular mortality in 205 482 people

with type 2 diabetes, stratified into four groups, depending on estimated glucose disposal rate (eGDR_BMI_)

| **Variable** | **eGDR_BMI_ (mg/kg/min)** | **Events**  **person-years** | **Rate per 1000**  **person-years**  **(95% CI)** | **Crude hazard ratio**  **(95% CI)** | **Age and sex adjusted hazard ratio (95% CI)** | **Multivariable adjusted^*^ hazard ratio (95% CI)** |
| --- | --- | --- | --- | --- | --- | --- |
| **Mortality** | **<4** | 783/5798 | 135.1 (125.8-144.9) | 1.00 | 1.00 | 1.00 |
|  | **4-6** | 1563/9601 | 162.8 (154.8-171.1) | 1.19 (1.09-1.30) | 0.91 (0.83-0.99) | 0.82 (0.70-0.94) |
|  | **6-8** | 633/3727 | 169.8 (156.9-183.6) | 1.24 (1.12-1.38) | 0.92 (0.83-1.03) | 0.75 (0.64-0.88) |
|  | **>8** | 253/1801 | 130.5 (114.3-148.3) | 0.99 (0.86-1.14) | 0.82 (0.70-0.94) | 0.68 (0.53-0.89) |
| **CV mortality** | **<4** | 421/5798 | 72.6 (65.8-79.9) | 1.00 | 1.00 | 1.00 |
|  | **4-6** | 851/9601 | 88.6 (82.8-94.8) | 1.20 (1.06-1.34) | 0.91 (0.81-1.03) | 0.82 (0.70-0.95) |
|  | **6-8** | 344/3727 | 92.3 (82.8-102.6) | 1.24 (1.07-1.43) | 0.91 (0.78-1.05) | 0.75 (0.60-0.93) |
|  | **>8** | 122/1801 | 67.7 (56.3-80.9) | 0.97 (0.80-1.19) | 0.80 (0.66-0.98) | 0.65 (0.45-0.93) |

*Multivariable adjusted (see text statistics) not adjusted for variables including in the eGDR formula, i.e. HbA1c, waist circumference

and blood pressure.

**Figure S1.** Spearman association curves (r-value) between M-values for the hyperinsulinemic clamp (X-axis) and estimated glucose disposal rate (y-axis) based on waist (eGDR_waist_) and BMI (eGDR_BMI_) top and bottom, respectively.

**Figure S2.** Flowchart for the studied group

416 247 patients with type 2 diabetes from the Swedish National Diabetes Register

322 562 subjects with type 2 diabetes from 2004 or later

199 016 subjects excluded because of missing data for any of the variables in the eGDR formula

303 713 subjects with type 2 diabetes from 2004 or later without a previous stroke

93 685 subjects excluded because of index year of type 2 diabetes before 2004

18 849 subjects excluded because of a previous stroke

98 231 subjects excluded because of missing data for any of the variables in the eGDR_BMI_ formula

205 482 subjects with type 2 diabetes with no missing data for eGDR_BMI_

104 697 subjects with type 2 diabetes with no missing data for eGDR

**Figure S3.** Adjusted hazard ratio (solid line) and 95% confidence intervals (dashed lines) for the association between baseline eGDR and stroke. The baseline eGDR level was modelled with restricted cubic splines in a Cox regression model adjusted for sex and age.


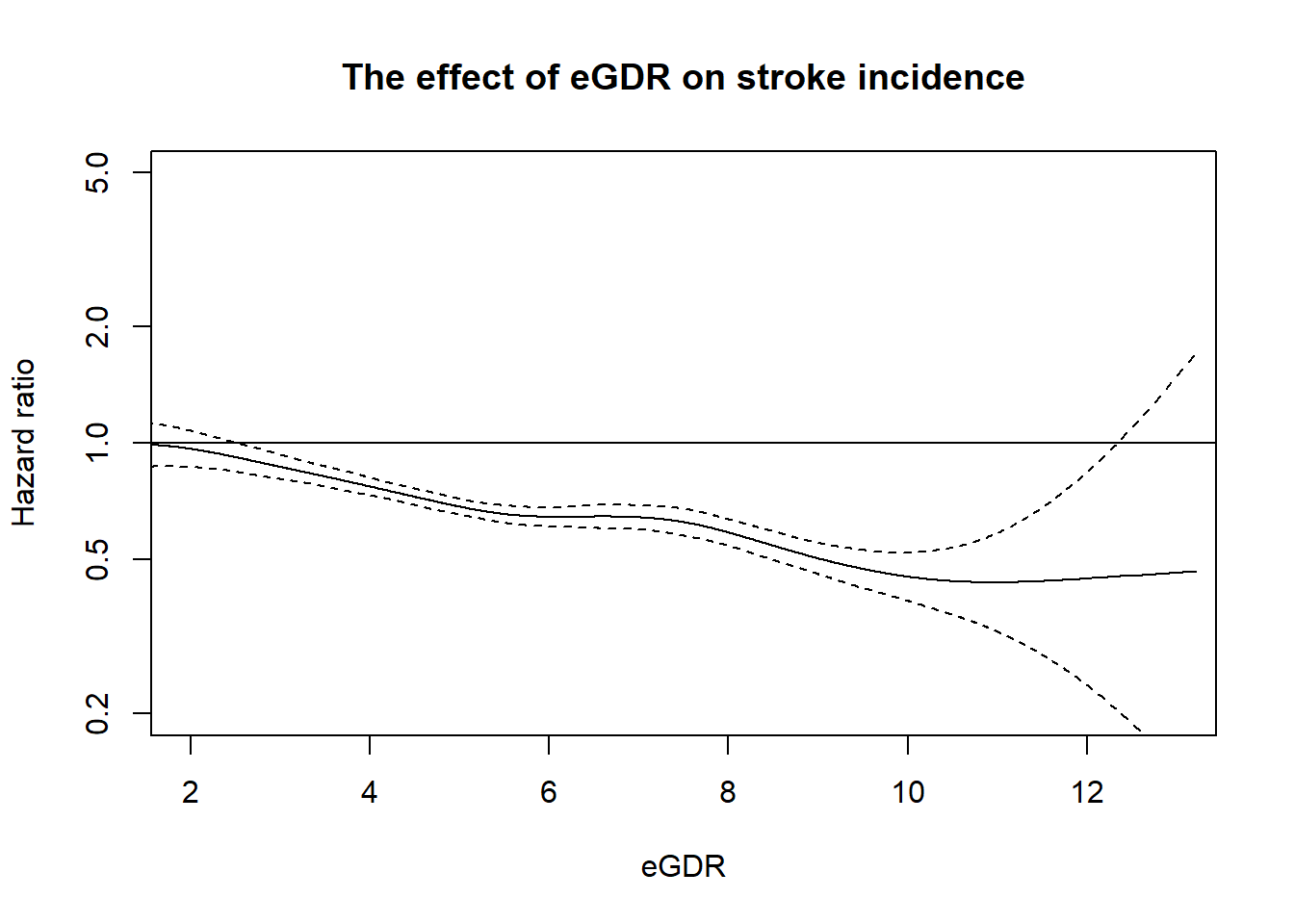


Figure S4. Fully adjusted hazard ratios for stroke; on insulin treatment and not on insulin treatment, divided into all stroke (ischaemic and haemorrhagic stroke), ischaemic and haemorrhagic stroke, respectively in 104 697 individuals with type 2 diabetes according to eGDR (Reference eGDR <4)

**
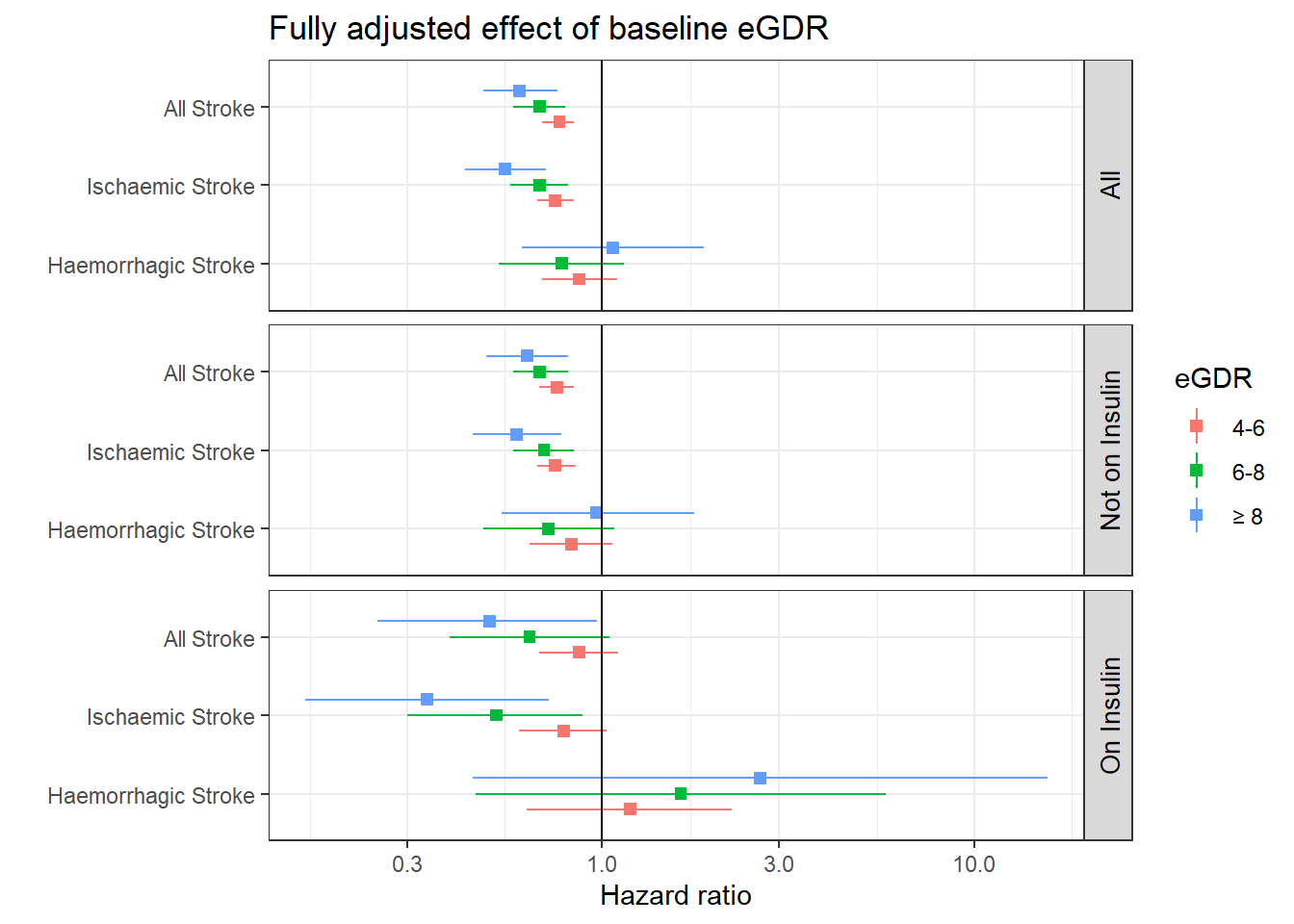
**

Figure S5. Cumulative incidence of stroke in in 205 482 patients with type 2 diabetes, divided into different groups depending on eGDR_BMI_


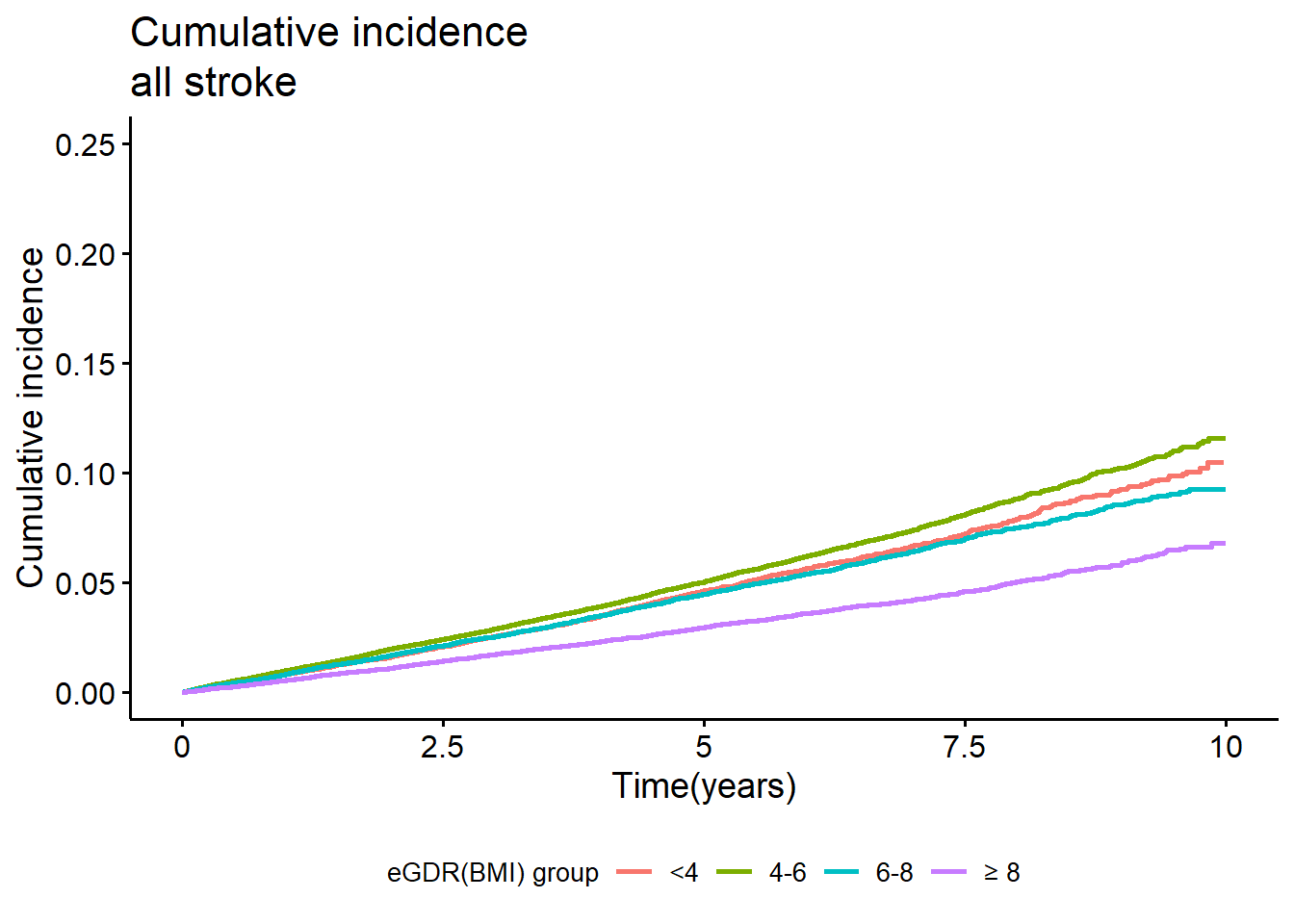


**Figure S6**. Hazard ratios for a first stroke, fully adjusted, divided into all stroke (ischaemic and haemorrhagic stroke), ischaemic and haemorrhagic stroke in 205 482 individuals with type 2 diabetes according to eGDR_BMI_ (Reference eGDR_BMI_ <4)


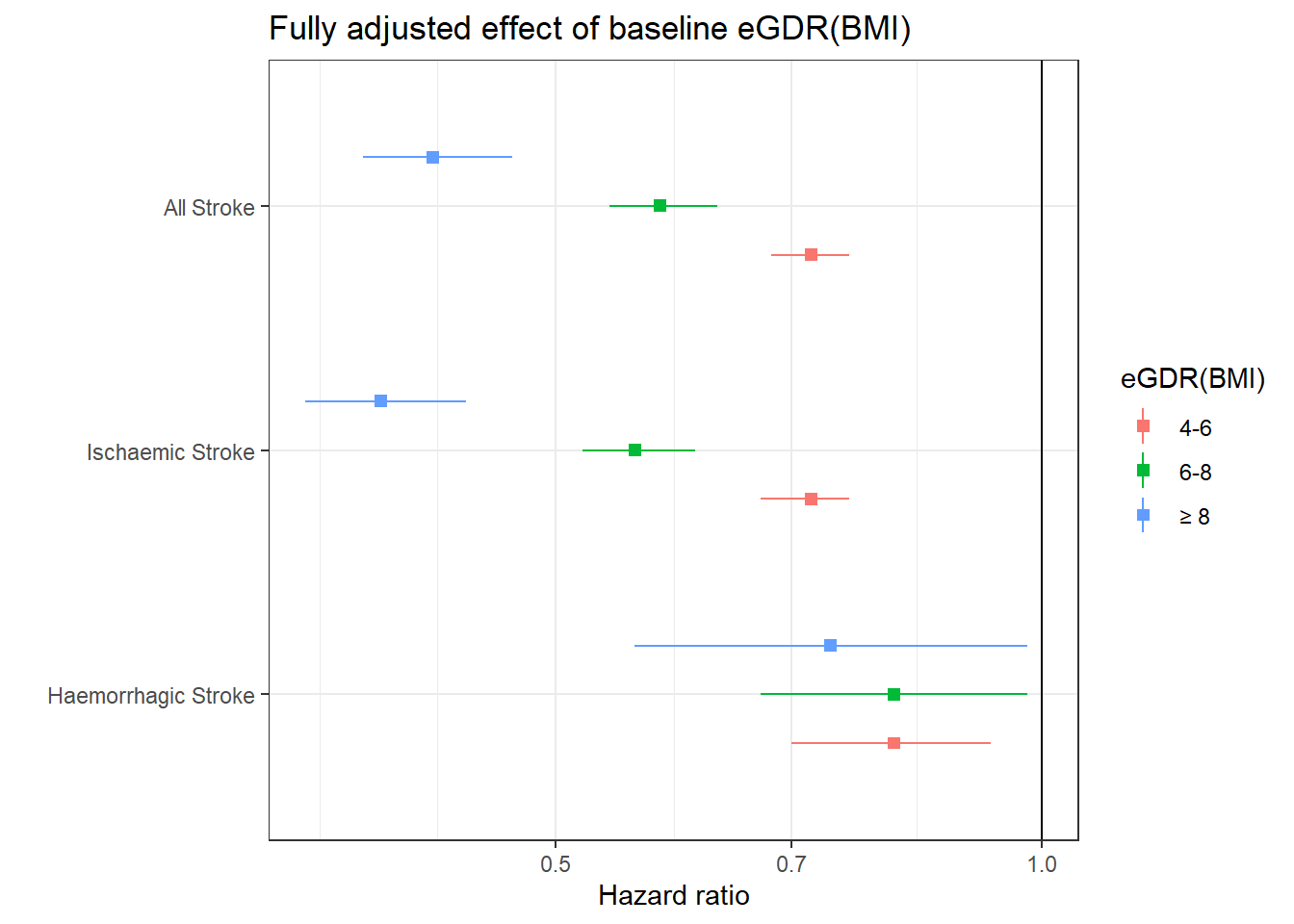


References

1. Williams KV, Erbey JR, Becker D, Arslanian S, Orchard TJ. Can clinical factors estimate insulin resistance in type 1 diabetes? Diabetes. 2000 Apr;49(4):626-32. doi: 10.2337/diabetes.49.4.626. PMID: 10871201.
2. Heller G. A measure of explained risk in the proportional hazards model. Biostatistics. 2012 Apr;13(2):315-25. doi: 10.1093/biostatistics/kxr047. Epub 2011 Dec 21. PMID: 22190711; PMCID: PMC3297826.

The RECORD statement **– checklist of items, extended from the STROBE statement, that should be reported in observational studies using routinely collected health data.**

|  | **Item No.** | **STROBE items** | **Location in manuscript where items are reported** | **RECORD items** | **Location in manuscript where items are reported** |
| --- | --- | --- | --- | --- | --- |
| **Title and abstract** | | | | | |
|  | 1 | (a) Indicate the study’s design with a commonly used term in the title or the abstract (b) Provide in the abstract an informative and balanced summary of what was done and what was found | Page 1 | RECORD 1.1: The type of data used should be specified in the title or abstract. When possible, the name of the databases used should be included.  RECORD 1.2: If applicable, the geographic region and timeframe within which the study took place should be reported in the title or abstract.  RECORD 1.3: If linkage between databases was conducted for the study, this should be clearly stated in the title or abstract. |  |
| **Introduction** | | | | | |
| Background rationale | 2 | Explain the scientific background and rationale for the investigation being reported | Page 2-3 |  |  |
| Objectives | 3 | State specific objectives, including any prespecified hypotheses | Page 3-4 |  |  |
| **Methods** | | | | | |
| Study Design | 4 | Present key elements of study design early in the paper | Page 4 |  |  |
| Setting | 5 | Describe the setting, locations, and relevant dates, including periods of recruitment, exposure, follow-up, and data collection | Page 4-5 |  |  |
| Participants | 6 | *(a) Cohort study* - Give the eligibility criteria, and the sources and methods of selection of participants. Describe methods of follow-up  *Case-control study* - Give the eligibility criteria, and the sources and methods of case ascertainment and control selection. Give the rationale for the choice of cases and controls  *Cross-sectional study* - Give the eligibility criteria, and the sources and methods of selection of participants  *(b) Cohort study* - For matched studies, give matching criteria and number of exposed and unexposed  *Case-control study* - For matched studies, give matching criteria and the number of controls per case | Page 4-5 | RECORD 6.1: The methods of study population selection (such as codes or algorithms used to identify subjects) should be listed in detail. If this is not possible, an explanation should be provided.  RECORD 6.2: Any validation studies of the codes or algorithms used to select the population should be referenced. If validation was conducted for this study and not published elsewhere, detailed methods and results should be provided.  RECORD 6.3: If the study involved linkage of databases, consider use of a flow diagram or other graphical display to demonstrate the data linkage process, including the number of individuals with linked data at each stage. |  |
| Variables | 7 | Clearly define all outcomes, exposures, predictors, potential confounders, and effect modifiers. Give diagnostic criteria, if applicable. | Page 5 | RECORD 7.1: A complete list of codes and algorithms used to classify exposures, outcomes, confounders, and effect modifiers should be provided. If these cannot be reported, an explanation should be provided. |  |
| Data sources/ measurement | 8 | For each variable of interest, give sources of data and details of methods of assessment (measurement).  Describe comparability of assessment methods if there is more than one group | Page 4-6 |  |  |
| Bias | 9 | Describe any efforts to address potential sources of bias | Page 4-6 |  |  |
| Study size | 10 | Explain how the study size was arrived at | Page 4 |  |  |
| Quantitative variables | 11 | Explain how quantitative variables were handled in the analyses. If applicable, describe which groupings were chosen, and why | Page 4-6 |  |  |
| Statistical methods | 12 | (a) Describe all statistical methods, including those used to control for confounding  (b) Describe any methods used to examine subgroups and interactions  (c) Explain how missing data were addressed  (d) *Cohort study* - If applicable, explain how loss to follow-up was addressed  *Case-control study* - If applicable, explain how matching of cases and controls was addressed  *Cross-sectional study* - If applicable, describe analytical methods taking account of sampling strategy  (e) Describe any sensitivity analyses | Page 6 |  |  |
| Data access and cleaning methods |  | .. | Page 4-6 | RECORD 12.1: Authors should describe the extent to which the investigators had access to the database population used to create the study population.  RECORD 12.2: Authors should provide information on the data cleaning methods used in the study. |  |
| Linkage |  | .. | Page 4 | RECORD 12.3: State whether the study included person-level, institutional-level, or other data linkage across two or more databases. The methods of linkage and methods of linkage quality evaluation should be provided. |  |
| **Results** | | | | | |
| Participants | 13 | (a) Report the numbers of individuals at each stage of the study (*e.g.*, numbers potentially eligible, examined for eligibility, confirmed eligible, included in the study, completing follow-up, and analysed)  (b) Give reasons for non-participation at each stage.  (c) Consider use of a flow diagram | a) Page 6 | RECORD 13.1: Describe in detail the selection of the persons included in the study (*i.e.,* study population selection) including filtering based on data quality, data availability and linkage. The selection of included persons can be described in the text and/or by means of the study flow diagram. |  |
| Descriptive data | 14 | (a) Give characteristics of study participants (*e.g.*, demographic, clinical, social) and information on exposures and potential confounders  (b) Indicate the number of participants with missing data for each variable of interest  (c) *Cohort study* - summarise follow-up time (*e.g.*, average and total amount) | a) Page 6  b) Page  c) Page 6 |  |  |
| Outcome data | 15 | *Cohort study* - Report numbers of outcome events or summary measures over time  *Case-control study* - Report numbers in each exposure category, or summary measures of exposure  *Cross-sectional study* - Report numbers of outcome events or summary measures | Page 6-7 |  |  |
| Main results | 16 | (a) Give unadjusted estimates and, if applicable, confounder-adjusted estimates and their precision (e.g., 95% confidence interval). Make clear which confounders were adjusted for and why they were included  (b) Report category boundaries when continuous variables were categorized  (c) If relevant, consider translating estimates of relative risk into absolute risk for a meaningful time period | a) Table 2  b) Page 6-8 |  |  |
| Other analyses | 17 | Report other analyses done—e.g., analyses of subgroups and interactions, and sensitivity analyses | N/A |  |  |
| **Discussion** | | | | | |
| Key results | 18 | Summarise key results with reference to study objectives | Page 8 |  |  |
| Limitations | 19 | Discuss limitations of the study, taking into account sources of potential bias or imprecision. Discuss both direction and magnitude of any potential bias | Page 12 | RECORD 19.1: Discuss the implications of using data that were not created or collected to answer the specific research question(s). Include discussion of misclassification bias, unmeasured confounding, missing data, and changing eligibility over time, as they pertain to the study being reported. |  |
| Interpretation | 20 | Give a cautious overall interpretation of results considering objectives, limitations, multiplicity of analyses, results from similar studies, and other relevant evidence | Page 12-13 |  |  |
| Generalisability | 21 | Discuss the generalisability (external validity) of the study results | Page 12-13 |  |  |
| **Other Information** | | | | | |
| Funding | 22 | Give the source of funding and the role of the funders for the present study and, if applicable, for the original study on which the present article is based | Page X |  |  |
| Accessibility of protocol, raw data, and programming code |  | .. |  | RECORD 22.1: Authors should provide information on how to access any supplemental information such as the study protocol, raw data, or programming code. |  |

*Reference: Benchimol EI, Smeeth L, Guttmann A, Harron K, Moher D, Petersen I, Sørensen HT, von Elm E, Langan SM, the RECORD Working Committee. The REporting of studies Conducted using Observational Routinely-collected health Data (RECORD) Statement. *PLoS Medicine* 2015;12(10):e1001885. DOI: 10.1371/journal.pmed.1001885. *Checklist is protected under Creative Commons Attribution ([CC BY](http://creativecommons.org/licenses/by/4.0/)) license.
